# miRNAs bridging the gap between the tumor microenvironment and liquid biopsy in cutaneous melanoma

**DOI:** 10.3389/fimmu.2026.1949578

**Published:** 2026-10-05

**Authors:** Monica Neagu, Elena-Georgiana Dobre, Carolina Constantin

**Affiliations:** 1 Immunology Laboratory, “Victor Babeş” National Institute of Pathology, Bucharest, Romania; 2 Department of Pathology, Colentina Clinical Hospital, Bucharest, Romania; 3 Doctoral School, Faculty of Biology, University of Bucharest, Bucharest, Romania

**Keywords:** circular RNA, cutaneous melanoma, exosomal miRNA, liquid biopsy, long noncoding RNA, miRNAs, molecular sponges

## Abstract

Cutaneous melanoma remains one of the most challenging neoplasms and the deadliest form of skin cancer. miRNAs are a class of non-coding RNA molecules found in both tissues and body fluids that can negatively regulate gene expression in eukaryotic cells, playing key roles in health and disease. Dysregulated miRNA levels have been linked to various aspects of melanocytic tumor biology, including the transition from benign to malignant stages, immunosuppression, dissemination, and therapy resistance. Here, we provide a timely review of the roles of miRNAs within the cutaneous melanoma tumor microenvironment, emphasizing their impact on immune cell phenotypes and functions, angiogenesis, innate and adaptive immunity, and immune checkpoint expression to help refine future biomarkers and therapeutic targets in the field. We will also address their roles in circular and long non-coding RNA sponging interactions and the potential for translating miRNAs into clinical practice, given their simultaneous presence in both cutaneous melanoma tissues and liquid biopsies. The main challenges and limitations associated with such a translational approach will also be addressed.

## 1 Introduction

Among skin cancers, cutaneous melanoma (CM) is a highly aggressive malignancy with an increased potential for metastasis. In skin cancers, unveiling the molecular mechanisms underlying aggressiveness is crucial for improving patient prognosis (1). Among the molecular players in CM, miRNAs play a seminal role in post-transcriptional mRNA regulation without encoding protein information. They are approximately 21–25 nucleotides in length and regulate messenger RNA (mRNA) by physically binding to it. This molecular interaction of miRNAs with mRNA induces mRNA degradation or functionally inhibits its translation into proteins, thus developing the process of gene silencing. Extensive miRNA studies have shown that these molecules are involved in tumor progression, cell proliferation, cell cycle, apoptosis, and tumor angiogenesis/invasion (2, 3).

In 2026, a distinct molecular signature of melanomas was described, spanning markers from genomic, epigenomic, transcriptomic, proteomic, and metabolomic domains identifiable in both tissue and blood circulation. These tissue markers can provide a precise diagnosis and forecast prognosis, and the markers investigated in blood and other biological fluids can monitor disease progression.

Circulating tumor DNA (ctDNA) enables the quantitative tracking of various mutations, including *BRAF*, *NRAS*, and *TERT* genes, using droplet digital PCR (ddPCR) and next-generation sequencing (NGS). ctDNA dynamics matches tissue genotypes, and when its profile is followed with the clinical evolution of patients, it can even anticipate imaging progression. Tissue and circulating miRNA signatures, such as the MEL38 panel, corroborate with different melanoma stages. Additionally, miRNA signatures, protein biomarkers, and metabolic profiling can aid prognostication and therapy monitoring in CM (4).

The specificity of miRNA profiling is high. A recent study performed on dysplastic nevi showed a distinct miRNA profile from melanoma compared to benign nevi. Hence, the study by Durante et al. suggests that dysplastic nevi are a distinct biological entity (5). In 2025, Geng et al. investigated Gene Expression Omnibus and The Cancer Genome Atlas (TCGA) databases regarding cutaneous melanoma (CM). They showed that a competing endogenous RNA (ceRNA) network comprising four mRNAs, 13 long non-coding RNAs (lncRNAs), and 20 miRNAs can be designed for CM. This network can identify new potential biomarkers for CM’s diagnosis, prognosis, and targeted immunotherapy (6).

Our review contemplates recent important findings regarding miRNAs within tissue and circulation and their correlations with various clinical frameworks of CM patients.

## 2 Tissue-based miRNA roles

As tumorigenesis is associated with major gene dysregulations, miRNAs and their gene-regulatory functions have recently attracted scientific attention regarding their role in cancer-associated alterations of gene expression. These studies focus on both research and future clinical application. Specific miRNAs detected in tumoral tissue can be prognostic and diagnostic markers and provide future therapeutic targets in cancers (7). In CM, non-coding RNAs (microRNAs, long non-coding RNAs, circular RNAs, and other small RNAs) are actively involved in tumor plasticity, progression, therapeutic response, and therapy resistance. Therefore, miRNAs can be promising biomarkers for diagnosis and prognosis (8) and for novel melanoma therapies (9), as further detailed below.

### 2.1 Diagnosis and prognosis through miRNA lenses

Over recent decades, changes in miRNA expression patterns in human cancers have driven investigations toward clarifying the mechanistic roles of miRNAs in cancer initiation and progression, as well as their power to be diagnostic and prognostic markers and future therapeutic targets. Melanoma is one of the tumors in which changes in miRNA expression may play a role in the progression of this skin cancer (10).

There are two main research streams in the microRNA (miRNA) domain, especially concerning melanoma. One investigates complex panels, while the other focuses on individual miRNAs correlated with various parameters. From the first branch, complex panels were studied in both primary CMs and various metastatic tissues. Their potential as diagnostic markers has been shown in various publications.

In a study published in 2024, Love et al. investigated disease-specific miRNA signatures in tissue and plasma. Patients were diagnosed with invasive melanoma or benign melanocytic proliferations. A specific 12-miRNA signature (MEL12) was identified as being able to stratify patients into low-, intermediate-, and high-risk groups and provided new information on therapeutic approaches (11). Results were validated by the same group in 2026 (12). Historically, MEL12 was preceded by the MEL38 pattern, published in 2019 by the same group. MEL38 consisted of 38 circulating miRNAs identified in CM patients (13).

Both miRNA signatures, MEL12 and MEL38, are valuable in diagnosis and prognosis. MEL38 classifies patients into diagnostic groups with a high predictive value for clinical status, while MEL12 stratifies patients into risk groups, independent of clinical covariates (**Figure 1**). The MEL38 and MEL12 signatures are functionally complementary but non-overlapping. **Table 1** depicts the actual patterns of MEL12 (39), and **Table 2** depicts the MEL38 panel (40), presented in the supplementary material.

**Figure 1:**
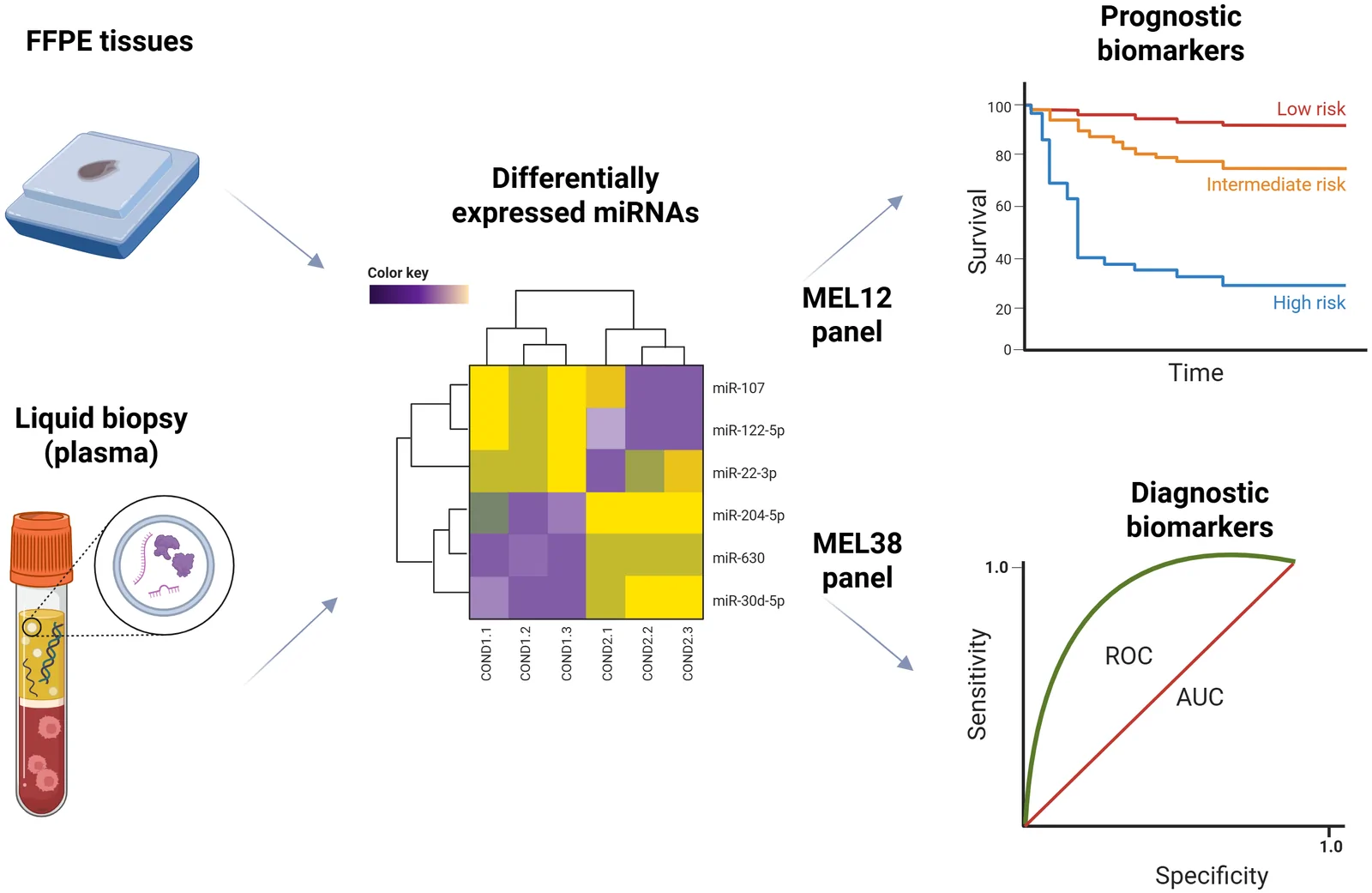
Clinical applicability of MEL12 and MEL38 miRNA panels in the diagnosis and prognosis of cutaneous melanoma patients. The two panels showed increased accuracy and robustness in both FFPE tissues and liquid biopsies. FFPE, formalin-fixed paraffin-embedded tissues; ROC, receiver operating characteristic curve; and AUC, area under curve. The figure was created in https://www.Biorender.com.

**Table 1:** Major sponging molecules lncRNA and circRNA in melanomas.

ncRNA	miRNA	Downstream target	Biological effect	Clinical correlations	Ref.
lncRNA					
MALAT1	miR-34a	*c-Myc*, Met transcripts	Up-regulated pro-tumorigenesis	Tumor growth, metastasis and drug resistance	(14)
	miR-183	ITGB1	Downregulation		(15)
	miR-22	MMP14, Snail transcription factors	Inhibition of the EMT expression of		(16)
MSC-AS1	miRNA-330-3p	YAP1	Upregulation in tissues and cell lines	Correlation with poor prognosis	(17)
*miRNA-155HG*	miRNA-485-3p	3’ UTR of *PSIP1*	Upregulation in tissues and cell lines	Decreased OS	(18)
*LINC01063*	miRNA-5194	*SOX12*	Pro-tumorigenenesis	Highly expressed in advanced melanoma stages	(19)
*LINC01291*	miRNA-625-5p	*IGF-1R*	Cell proliferation, colony formation, migration, and invasion	Future therapeutic target for melanoma	(20)
*LINC01116*	miRNA-432-5p	*FKBP7/14*	Proliferation, spheroid formation, and invasiveness	Diagnostic biomarker	(21)
*LINC00847*	miRNA-133a-3p	*TGFBR1*	Up-regulation in tissue and cell lines	Low expression in benign nevi	(22)
*LINC00662*	miRNA-107	*POU3F2*	Up-regulation in tissues	Melanoma progression	(23)
*LINC01296*	miRNA-324-3p/	*MAPK1*	Aggressive tumor behavior		(24)
*LINC00518*	miRNA-526b-3p	*EIF5A2*	Proliferation, invasion, and migration	Correlated with decreased survival	(25)
*LINC01224*	miRNA-193a-5p	*NR1D2*	Oncogenic effect	Upon knock-down, it restores radiosensitivity	(26)
*KCNQ1OT1*	miRNA-34a	*STAT3*	Immune evasion of melanoma cells	Effect on PD-L1	(27)
*HCG18*	miRNA-324-5p	*CDK16*	Proliferation, migration, cell cycle, and apoptosis	Marker for diagnosis	(28)
*DANCR*	miRNA-5194	*VEGFB*	Enhanced angiogenesis	Therapy target	(29)
*SNHG8*	miRNA-656	*SERBP1*	Viability, migration, and invasion	Therapy target	(30)
*CYTOR*	miRNA-485-5p	*GPI*	Proliferation, migration, and invasion	Correlated with reduced OS	(31)
*MEG3*	miRNA-208	*SOX4*	Upregulation suppresses proliferation and metastasis	Therapy target	(32)
circRNAs					
*hsa_circ_0002490*	RNABP	*DND1*	Tumor growth, migration, and invasion	Correlated with reduced OS	(33)
*circRTTN*	miRNA-890	*EPHA2*	Melanoma progression		(34)
*CircFAT1*	miRNA-375	*SLC7A11*	Invasion and proliferation		(35)
*Hsa_circ_0103232*	miRNA-661	*RAB3D*	Tumor promoter		(36)
*CircRPS5*	miRNA-151a	*NPTX1*	Suppressor of proliferation, migration, invasion, and inducer cell cycle arrest	Therapy strategy	(37)
circITCH,	miRNA-660	*TFCP2*	Downregulation of proliferation and metastasis	Therapy strategy	(38)

ITGB1, integrin subunit beta 1; MSC-AS1, MSC antisense RNA 1; GPI, glucose-6-phosphate isomerase; DND1, dead end protein homolog 1; RNABP, RNA binding protein sites; EPHA2, EPH receptor A2; SLC7A11, solute carrier family 7 member 11; RAB3D, GTPase regulating intracellular membrane trafficking; NPTX1, neuronal pentraxin 1; and TFCP2, transcription factor CP2.

**Table 2:** miRNAs that comprise MEL38 panel for melanomas.

MicroRNA	Target	Action - involved in
hsa-miR 424-5p	LATS2 gene	Angiogenesis/inflammation
hsa-miR 548l		Invasion/metastasis
hsa-miR 34a-5p	ZEB1	Drug resistance and metastasis
hsa-miR 497-5p	hTERT	Tumour suppressor, Angiogenesis/inflammation
hsa-miR 299-3p	VGFA	Invasion/metastasis
hsa-miR 205-5p	TP53, NFKB	Tumour suppressor
hsa-miR 1269a	SMAD4	Tumour suppressor
hsa-miR 624-3p	NFKB1, PTEN	Drug resistance
hsa-miR 138-5p	NFKB1, Trp53	Invasion/metastasis
hsa-miR-1 5p	NRAS, TLR, NF-κB, ErB paths	Invasion/metastasis and drug resistance
hsa-miR 152-3p	CDK6	Drug resistance
hsa-miR 1910-5p	CCL20, TGFBR2	Invasion/metastasis
hsa-miR 181b-5p	CDTSPL	Tumour suppressor/oncogene
hsa-miR 3928-3p	CCL3/CCR5 axes	Invasion/metastasis
hsa-miR 3131	DMNT, KRAS	Invasion/metastasis
hsa-miR 301a-3p	multiple genes, e.g. SPRED1, VCAM1, ADGRE5, CXCL12	Invasion/metastasis
hsa-miR 1973	ARMC8	Drug resistance
hsa-miR 520d-3p	ASF1B	Tumour suppressor/ oncogene
hsa-miR 548a-5p		Tumour suppressor/oncogene
hsa-miR 548ad-3p		Tumour suppressor/oncogene
hsa-miR 454-3p		Drug resistance
hsa-miR 4532	NFKB1	Drug resistance
hsa-miR 1537-3p		Angiogenesis/inflammation
hsa-miR-553	NFKB1	Tumour suppressor/ oncogene
hsa-miR-764	TP53 and NFKB1	Invasion/metastasis
hsa-miR 1302		Invasion/metastasis
hsa-miR 1258	TP53, EGFR, SMAD2	Invasion/metastasis
hsa-miR 522-3p	HMGB1	Tumour suppressor/oncogene
hsa-miR 1264	TP53, DNMT1	Drug resistance
hsa-miR 1306-5p	mTOR	Tumour suppressor/oncogene
hsa-miR 219a-2-3p	TERT	Drug resistance
hsa-miR 431-5p	XIAP, SMAD4	Drug resistance
hsa-miR 450a-5p	CXCR4, EGFR	Invasion/metastasis
hsa-miR 2682-5p		Drug resistance
hsa-miR 337-5p	ERBB2, VEGF2	Invasion/metastasis
hsa-miR 27a-3p	FZD7, TP53 and NFKB1	Invasion/metastasis
hsa-miR 4787-3p		Invasion/metastasis
hsa-miR 154-5p	AGTR1	Invasion/metastasis

Moreover, in **Figure 2**, we have depicted the individual miRNAs from the MEL12 and MEL38 panels, classified as tumor suppressors or having oncogenic functions, and even those with dual functions. From **Figure 2**, it can be observed that, at least from these two complex panels, the majority of miRNAs have pro-tumorigenic functions.

**Figure 2:**
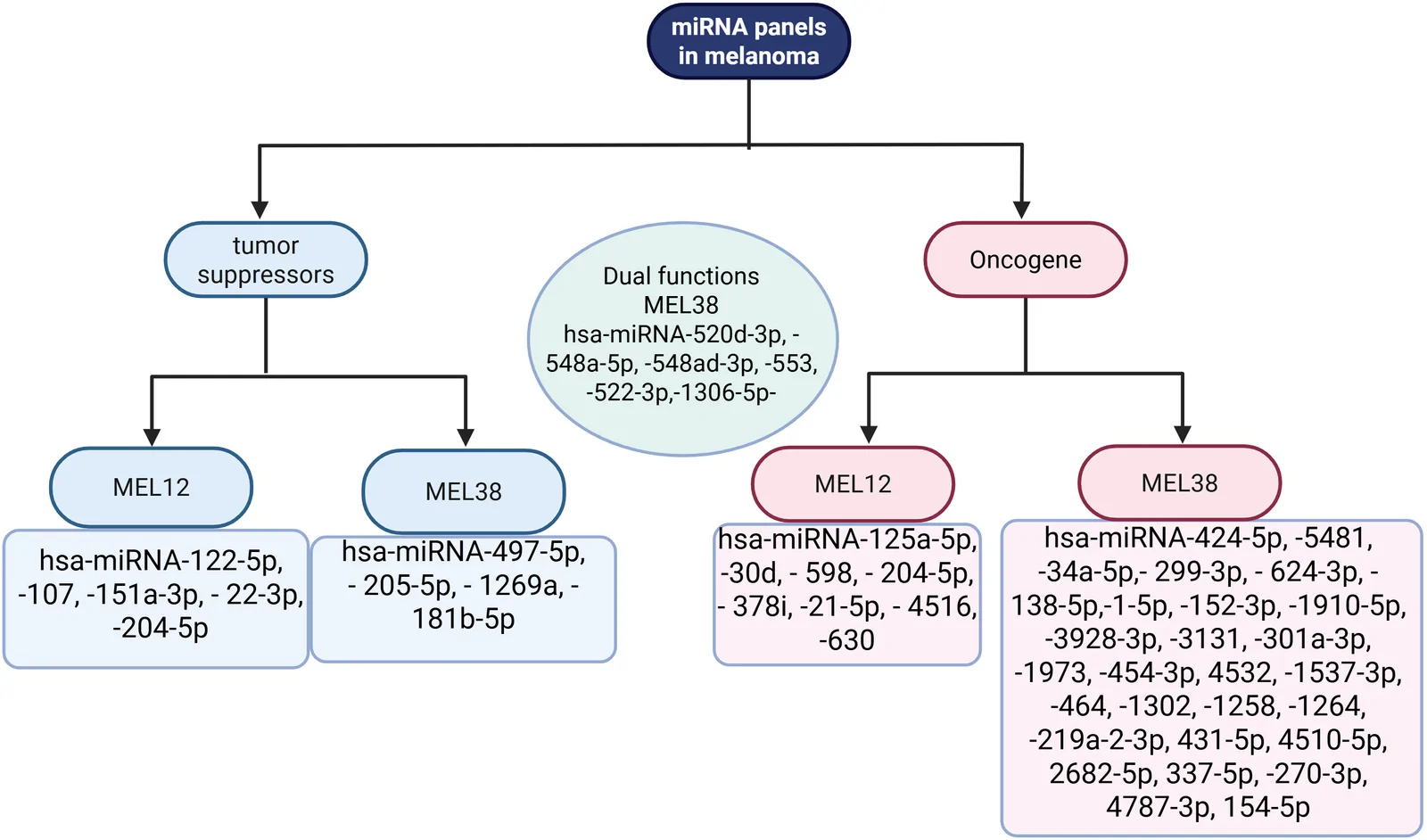
MEL12 and MEL38 panels detailed on their functions: tumor supressors, oncogene or dual functions. The figure was created in https://www.Biorender.com.

The MEL38 and MEL12 panels, initially identified as circulating biomarkers, were further found to be reproducible and robust in formalin-fixed paraffin-embedded (FFPE) tissues. Thus, when applied to a clinical cohort of 61 FFPE and 59 plasma specimens, the diagnostic panel MEL38 distinguished CM from normal subjects with an area under the curve (AUC) of 0.99 in plasma and 1 in FFPE. In addition, the MEL12 panel proved effective in stratifying patients into risk groups, indicating a 1.7-fold higher risk of death in intermediate- versus low-risk groups and a 2.3-fold higher risk of death in high-risk compared to intermediate-risk patients. MEL12 outperformed clinicopathological variables and other traditional genomic biomarkers in predicting outcomes in multiple clinical cohorts and in the TCGA-skin cutaneous melanoma (SKCM) dataset, highlighting its suitability in CM precision oncology (12, 13).

Other differentially expressed miRNAs between primary and metastatic melanomas may also serve as companions for these two complex panels. These include tumor-suppressive miRNAs such as miR-205 and members from the miR-200 and miR-203 families, and the onco-miRNA 675-3p (41–43). For instance, studies of clinical cohorts show that the loss of miR-205 and miR-200a, alongside increased miR-675-3p, characterize metastatic melanoma and have been linked to aggressive features and poor overall survival (OS) (43–45).

Furthermore, miRNA panels studied by Di Vincenzo et al. have shown a specific profile of ulcerated CM. Using Nanostring human miRNA and tumor signaling 360 mRNA assays, it was proven that ulcerated melanomas have at least a 1.5-fold change in the expression of 24 miRs (e.g., miR-206, miR-1-3p, miR-4286, miR-146a-5p, miR-196b-5p, and miR-363-3p). Moreover, ulcerated tumors have 21 differentially expressed mRNAs compared to non-ulcerated tumors, while two are inversely correlated: the mRNA for *SOCS3* with miR-218-5p, and the mRNA for *IL7R* with miR-376c-5p (46).

Wiggins et al. studied miRNA expression in patients with stage II and stage III resected melanomas to integrate them into a prognostic model. Using a Nanostring miRNA expression assay to analyze over 700 primary melanomas from stage II or stage III patients, an association between miRNA expression and melanoma clinical outcome was found. The authors report 15 candidate miRNAs associated with 5-year survival for stage II, while eight candidate miRNAs were identified for stage III. Two miRNAs, miR-150–5p and miR-142–3p, were common to both stages. Integrating tissue miRNA patterns within the prognostic landscape would enhance the accuracy of clinical prognostics (47).

Apart from the presented panels, individual miRNA expression could aid the molecular characterization of melanoma tissue. Knowing that gene mutations induced by ultraviolet (UV) radiation exposure are important triggers in CM, various pathways triggered upon UV exposure were studied. Therefore, redox-mediated signaling that alters miRNA profiles can induce CM initiation and progression. This UV-dependent redox regulation interferes with miRNA biogenesis and processing, interferes with redox-sensitive transcription factors, and with the induction of epigenetic modifiers (48). Individual miRNAs have been studied by various groups. For example, Fawzy et al. found miR-155 upregulation in over 100 patients with both primary and metastatic melanomas. When comparing miR-155 expression across malignant stages, it was found to be overexpressed in primary melanomas and precancerous lesions. The increased expression of miR-155 was also associated with superficial spreading melanoma and melanomas with a high mitotic rate. The study concludes that miR-155 could have potential prognostic utility in CM (49).

The miRNA landscape in melanoma tissue comprises both oncogenic miRNAs (e.g., miR-18a-5p, miR-146a) and tumor-suppressive miRNAs like miR-128-3p (3, 50). miR-876 is encoded by genes that commonly reside within *CDKN2A* on chromosome 9p21. *CDKN2A* functions as a tumor suppressor gene and frequently drives melanoma progression (51). miR-876 expression was found to be significantly decreased in primary melanomas when compared with nevi. Analyzing TCGA database deletions in miR-876, it was noted that in over 50% of CM cases, its expression was decreased. Another individual miRNA, miR-582, was found to be significantly decreased in both melanoma cell lines and tissues and further associated with ulceration, lymph node metastasis, TNM stage, and clinically with poor OS. The target of miR-582 is *FOXC1* (forkhead family of transcription factors), known to be involved in regulating various cellular and developmental processes. Overall, miR-582 may be a prognostic biomarker and a new therapeutic target for melanoma (52).

Although numerous studies to date attest to the accuracy of various individual miRNAs in predicting prognosis and treatment response in melanoma, the complexity of the disease necessitates the use of broader biomarker panels to obtain a more detailed overview of the deregulated biological processes within the tumor.

#### 2.1.1 miRNAs in melanoma metastasis

As CM is a highly metastatic tumor, and its metastases can have a different genetic makeup (53), several miRNAs have been investigated in metastatic tissues and found to correlate with this process (54). In particular, miRNA-181a/b was found in the metastasis variants of thin and thick primary CMs, highlighting its potential as a predictive biomarker (44). More than 50% of melanoma metastases occur in the brain, and Moubarak et al. have shown that miRNA-124a functions as a suppressor of melanoma brain metastasis. This can be therapeutically leveraged to impair metastasis in melanoma (55). A panel of six miRNAs (hsa-miR-141-3p, hsa-miR-141-5p, hsa-miR-146a-5p, hsa-miR-194-5p, hsa-miR-200b-3p, and hsa-miR-365b-5p) could discriminate brain metastases with CM as primary tumors. The study is important for stratifying brain metastases of unknown primary origin (56). Brain metastasis from an unknown primary tumor is a significant medical challenge, as this issue can frequently occur in melanoma if the primary CM was misdiagnosed or if there was no histological diagnosis of the excised tumor (57).

The first study published in 2025 by Ruckova et al., comparing miRNAs evaluated in metastatic tissue, cerebrospinal fluid (CSF), and plasma, has shown that melanoma brain metastases have miRNA enrichment pertaining to the regulation of cell cycle and nucleocytoplasmic transport. The results indicate that non-specific miRNAs circulating in the peripheral blood (e.g., miRNA-16-5p and miRNA-486-5p from the erythroid-specific group) complicate data interpretation and may hinder the detection of tumor-derived miRNAs (58). In over 100 samples of melanoma brain metastasis, miRNA-211-5p was evaluated, showing that high miRNA-211-5p expression is more frequent in primary tumors compared to paired metastases and is associated with worse OS. Therefore, the expression of miRNA-211-5p in brain metastasis can be a risk predictor in melanomas (59).

β-tubulin 4A (TUBB4A) expression within the tissue is known to be positively correlated with the OS of CM patients. The genes co-expressed with TUBB4A were enriched in melanoma-related pathways and functions (60). Investigating seven databases (e.g., PITA, miRmap, miRanda, RNA22, microT, PicTar, TargetScan) to predict miRNAs that might bind to *TUBB4A*, 25 miRNAs were found to bind to it. Out of these, high expression of hsa-let-7b-5p and hsa-mir-423-5p correlated with OS, while hsa-let-7c-5p, hsa-mir-18a-5p, and hsa-mir-493-3p correlated with poor OS (61). These data led to the conclusion that *TUBB4A* expression is positively correlated with OS in melanoma patients and may be a prognostic marker (60). miRNAs involved in the regulatory axes of the hsa-let-7b-5p-*TUBB4A* pathway may also be linked with CM progression (61).

As expected, miRNAs regulate transcription factors in melanomas. Abnormal expression of the RUNX family transcription factor 3 (*RUNX3*) has been reported in solid cancers, including melanomas. As *RUNX3* expression was found to be significantly upregulated in CM, its miRNA regulators were recently researched. Over 30 miRNAs target *RUNX3*, with miR-326, miR-330-5p, and miR-373-3p as examples (62).

All these presented interactions governed by miRNAs can themselves become new biomarkers for melanoma tissues, whether as diagnostic/prognostic biomarkers or even therapy targets.

### 2.2 Tumor microenvironment modulation

#### 2.2.1 miRNAs regulate CM sensitivity to targeted therapy

The presence of *BRAF* mutations that hyperactivate MAPK signaling represents the most commonly exploited therapeutic vulnerability in CM, enabling the design of *BRAF* and MAPK inhibitors. These inhibitors have significantly improved survival in clinical cohorts; however, a significant proportion of patients develop resistance to targeted therapy through *MAPK* reactivation or bypass pathway (PI3K/AKT) induction, metabolic reprogramming, and tumor microenvironment (TME) remodeling (63). A relatively recent study using FFPE tissues revealed elevated expression of miRNA-146a-5p and miRNA-4286 in melanoma tissues, particularly in *BRAF V600*-mutant samples. Specifically, miRNA-146a-5p modulates a low-grade inflammatory environment favorable to melanoma, while miRNA-4286 enhances cellular proliferation and inhibits apoptosis. Together with MAPK signaling, this creates a profile that supports tumor development and immune evasion in *BRAF V600E*-positive melanomas. Therefore, given that miRNA-146a-5p and miRNA-4286 are considered significant modulators of the oncogenic process driven by *BRAF* mutations, a more detailed evaluation of their tissue expression in patients resistant to *BRAF* inhibitors could reveal new mechanisms for sensitivity restoration in clinical settings (64–66).

As miRNAs are critical regulators in the tumor milieu and melanoma progression, they can be good therapeutic targets within the TME. *In vitro* manipulation of melanoma cells to upregulate miRNA-876 led to the abolishment of its pro-tumorigenic function. Moreover, computational analyses showed that *MAPK1/ERK2* were the targets of miRNA-876 interference. Bezrookove et al. suggested that combining miRNA-876 induction with *BRAF* inhibition could be a novel therapeutic strategy for melanoma (67).

Additionally, miRNA-224-5p was found to be downregulated in melanoma samples and identified as an independent risk factor for melanoma patients. miRNA-224-5p is involved in the inhibition of proliferation and invasion, and this miRNA can overcome acquired BRAFi resistance in melanoma cells. miRNA-224-5p directly represses *PAK4*, blocking the downstream *CRAF/MEK/ERK* pathways, and is involved in the attenuation of CM metastatic potential (68).

Understanding the role of non-coding RNAs (ncRNAs) in melanoma growth driven by *BRAF* mutations and treatment resistance is becoming crucial for the development of new therapeutic approaches. The relationship between key ncRNAs and *BRAF* mutations in melanoma was recently reviewed by Afsar et al., highlighting that miR-15/16 and miR-200 modulate Wnt signaling and melanogenesis. Moreover, miRNA-579-3p and miRNA-1246 are ncRNAs involved in drug resistance, autophagy, and immune checkpoint resistance (69).

#### 2.2.2 miRNAs are involved in CM aggressiveness

Disruption of the extracellular matrix (ECM) by specific proteases is a prerequisite for melanoma metastasis. This ECM turnover is linked not only to melanoma cell growth and invasion but also to the inflection of melanoma immunogenicity. Complex regulatory mechanisms at both the transcriptional and post-transcriptional levels impact these fluctuations in ECM protease activities. At the epigenetic level, miRNAs are regulators that can directly target ECM protease transcripts and influence the activity of ECM proteases and melanoma progression. Moreover, gut dysbiosis appears to induce changes in miRNA profiles targeting these proteases in melanoma progression. All these pieces of evidence could form the foundation for developing innovative strategies aimed at overcoming resistance to immunotherapies (70, 71).

Fibroblasts in melanoma’s TME are important players in the metastatic process, as cancer-associated fibroblasts (CAFs) shape the TME and organize the pre-metastatic niche. MiRNA-141-3p is associated with fibroblast growth factor 13 (*FGF13*) and the *MAPK* pathway. In melanoma tissues, *FGF13* is highly expressed, while miRNA-141-3p was found to be underexpressed. Further analysis highlighted *FGF13* as a target gene of miRNA-141-3p. Overexpression of miRNA-141-3p reduced the proliferative, invasive, and migratory potential of melanoma cells. This overexpression increased apoptosis *via* the disruption of the *FGF13*-dependent *MAPK* pathway (72).

Epithelial-mesenchymal transition (EMT) is a crucial cellular process for cancer progression and metastasis (73, 74) and a seminal process that modulates the TME niche. EMT-related genes like *LGALS1*, *CPXM1*, and *APLNR* were found to be overexpressed in melanomas, and of these, *LGALS1* was identified as a target for miRNA-22-3p. When miRNA-22-3p binds to *LGALS1*, the inhibition of melanoma cell EMT is triggered (75). EMT-related factors could be altered by the overexpression of miRNA-498 in melanoma cells through directly targeting *UBE2T* 3’-UTR and inhibiting its expression. In CM patients, high expression of *UBE2T* was associated with poor OS and increased metastasis (76). Poor OS in melanoma patients was also observed when *circANKRD52* was overexpressed in melanoma samples. Knocking down *circANKRD52* in experimental models repressed growth and invasion. *circANKRD52* interacted with miRNA-141-3p, upregulating *PRKACB* and inducing the invasive and angiogenic potential of tumor cells (77).

Several other miRNAs may also modulate angiogenesis to help melanoma cells survive and spread to secondary sites. MiRNA-374b-5p was found to be a tumor suppressor in melanoma; thus, in a B16F10 murine model, it was demonstrated that the overexpression of miRNA-374b-5p significantly reduced tumor growth and angiogenesis and downregulated the lymphangiogenic factor *VEGFC*. In addition, overexpression of miRNA-374b-5p is also associated with changes in inflammatory markers related to macrophages, suggesting a shift toward a more pro-inflammatory macrophage phenotype. These findings propose miRNA-374b-5p as a new therapeutic target and a novel regulator of melanoma progression that acts through *VEGFC*-associated *MAPK* signaling and TME reprogramming. This recent study not only illustrates the multiple antitumor functions of miRNA-374b-5p in melanoma but also creates a conceptual framework for exploring miR-374b-5p-based strategies in melanoma therapy, such as combining miR-374b-5p approaches with anti-angiogenic agents or immune checkpoint inhibitors (78).

#### 2.2.3 miRNAs modulate immune checkpoint expression within the TME

Immune checkpoint inhibitors (ICIs) against the programmed cell death 1 (PDCD1/PD1) receptor and programmed cell death-ligand 1 (CD274/PD-L1) were a therapeutic breakthrough in melanoma. Despite this breakthrough, therapy resistance appears in a significant percentage of melanoma patients, and the TME is a site that governs this process (79). Six miRNAs targeting the 3’-UTR regions of *PD-L1* were identified (e.g., miR-26a/b-5p, miR-29a-3p, miR-103b, miR-181b-5p, miR-186-5p). The miRNA-mediated inhibition of *CD274* expression increased T-cell-specific recognition. The study by Vaxevanis et al. designed a *CD274* miRNA score associated with disease progression and reduced OS, providing new tools to develop miRNA-based therapies in melanoma (80). *CD274*-regulating miRNAs may be one of the future therapeutic approaches in CM (80). In the immunosuppressive TME, there are a myriad of miRNAs mediating a critical bidirectional connection between tumor cells and CD8+T cells. In the TME, miRNA involvement is complex; they regulate melanoma growth: hypoxia-induced immune escape and PD-1/PD-L1 checkpoint expression (miRNA-210, miRNA-192-5p, miRNA-34a), essential enhancers of T-cell-mediated antitumor immunity (miRNA-155, miRNA-25-3p/155-5p/215-5p/375, hsa-miRNA-320a-3p/mmu-miR-7037-5p/666-3p) (81, 82), and bidirectional regulators of the adaptive (miRNA-155 and miRNA-146a) and innate immune systems (miRNA-23a/24a/27a) (83).

#### 2.2.4 miRNAs regulate mitochondrial metabolism players and cell surface receptors

There are additional miRNA interactions in CM tissues that could serve as diagnostic/prognostic markers or even new therapeutic targets. In melanoma cell lines and tissues, the interaction between miRNA-210 and *ISCU* (iron-sulfur cluster assembly enzyme ISCU, mitochondrial) was studied. miRNA-210 was found to be upregulated in melanoma tissues and under hypoxic conditions. The *ISCU* gene was identified as a direct target of miRNA-210, and the authors contend that the miRNA-210/*ISCU*/ROS axis can serve as a novel therapeutic target in CM (84). Moreover, miRNAs can interact with cellular receptors, regulating their functions. For example, in a recent experimental design, the role of miRNA-425-5p in the context of melanoma malignancy was investigated. Thus, it was postulated that molecular mechanisms underlying miRNA-425-5p-mediated regulation of malignant melanoma proliferation and metastasis may include its interaction with scavenger receptor class A member 5 (*SCARA5*); this receptor acts as a tumor suppressor in multiple cancer types and has been reported to inhibit melanoma cell proliferation and migration by inducing ferroptosis (85). Moreover, decreased *SCARA5* expression was linked to immune infiltration levels and associated with a poor melanoma prognosis (86). In A375 and A2058 melanoma cell lines, it was revealed that inhibition of miRNA-425-5p would increase *SCARA5* expression and further repress cellular proliferation and metastasis. *In vivo*, in a nude mouse tumor model, although *SCARA5* overexpression significantly suppressed tumor growth, administration of miRNA-425-5p agonists demonstrated an adverse effect by supporting tumorigenesis. Thus, proteomics data revealed that miRNA-425-5p negatively regulates *SCARA5* expression, inhibiting apoptosis, activating AKT phosphorylation, and inducing epithelial-mesenchymal transition (EMT), thereby promoting melanoma progression. These data endorse the miRNA-425-5p/*SCARA5* axis as a possible therapeutic target for melanoma (87).

In recent years, miRNAs have proven to be important players in the therapeutic domain and can open new avenues for additional therapy in CM. Collectively, these miRNA-mediated interactions provide a framework for developing new combination therapies and improving precision immunotherapies (88). Since miRNAs can modulate the expression of immune checkpoints, influencing antitumor immune responses in melanoma, it is mandatory to further explore the potential of microRNAs as diagnostic markers, prognostic indicators, and predictors of therapeutic response. Deciphering the complex networks of microRNAs within the tumor milieu would provide new viewpoints for precision medicine in melanoma therapy (89).

### 2.3 Sponging networks as regulators of miRNAs in melanoma

#### 2.3.1 lncRNAs

Various important interactions of miRNAs have been identified in melanoma tissues. One of the most studied mechanisms is the interplay between long non-coding RNA (lncRNA) and miRNAs, a mechanism known as sponging.

The sponging process, recently discovered, highlights the fact that lncRNAs can compete with miRNAs for mRNA binding, thereby reducing their regulatory effect (90). A general scheme of interaction is presented in **Figure 3**. In CM, lncRNAs can guide phenotype switching and initiate metastasis, or they can favor therapy-resistant phenotypes. As biomarkers, the presence of lncRNAs within the tissue can predict therapy response in patients (91). A recent comprehensive review on lncRNAs has shown the numerous functions of these miRNA regulators (92).

**Figure 3:**
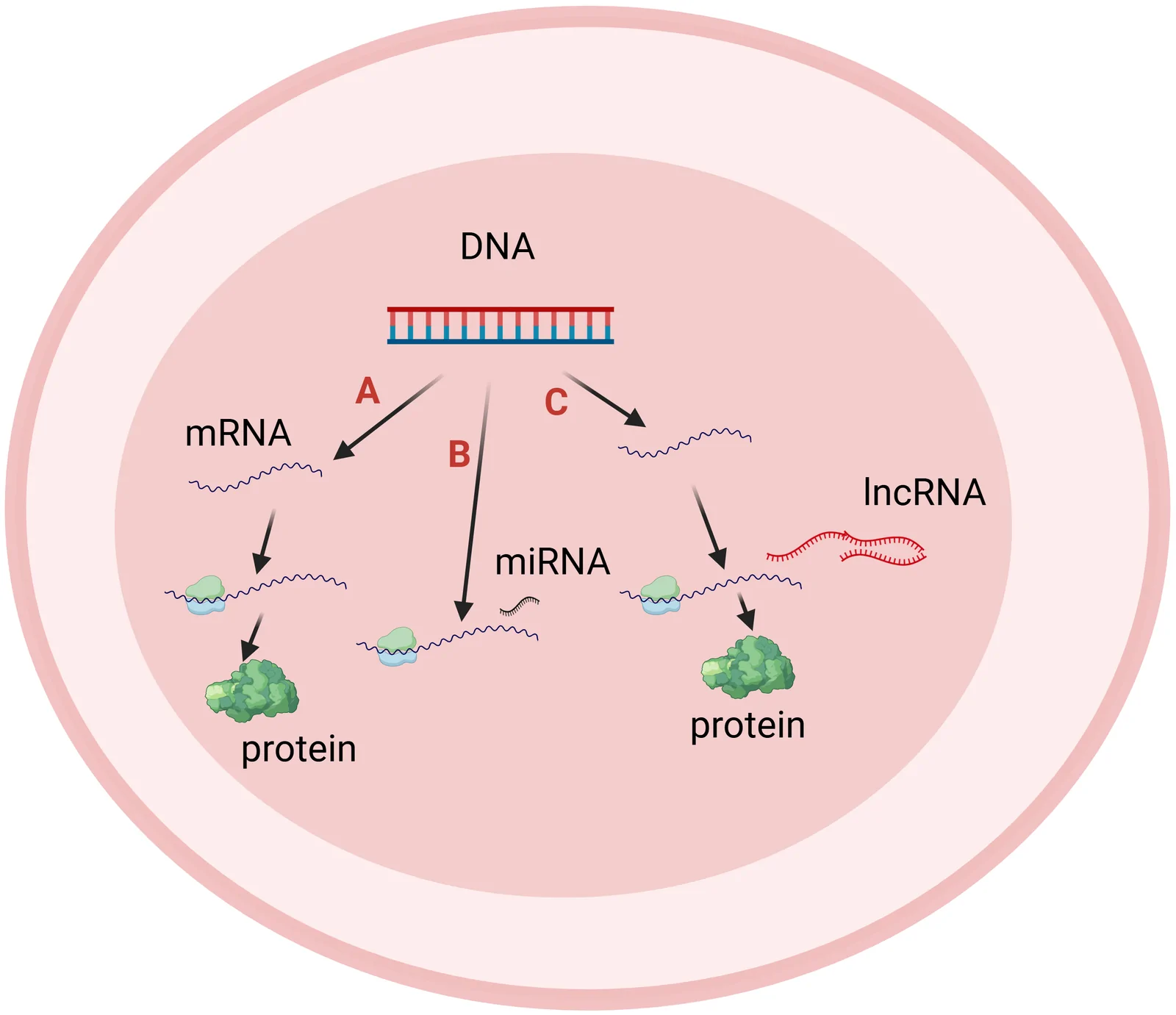
Sponging mechanisms that regulate miRNA functions in cells. **(A)** When mRNA translates directly to generating proteins; **(B)** When miRNA interferes with the translations, no protein is generated; **(C)** when lncRNA interferes with miRNA translations, it leads to protein generation. The figure was created in https://www.Biorender.com.

There are extensive studies regarding lncRNA interactions with miRNAs and their effect within melanoma tumor tissues. Moreover, lncRNAs can serve as precursors of miRNAs or circular (circ)RNAs (93). An earlier study by Iyer et al. identified 339 lncRNAs associated with this skin cancer in melanomas (94). Out of these, we will highlight a few that are more closely linked to clinical relevance in diagnosis/prognosis and possible therapy targets in CM.

Zhu et al. identified three key LincRNAs involved in melanoma development and progression: metastasis-associated lung adenocarcinoma transcript 1 (*MALAT1*), *LINC00943*, and *LINC00261* (95). *MALAT1* has a complex network of involvement in melanomas, regulating gene and protein expression in the *MAPK* pathway. The impact of antisense oligonucleotide (ASO)-based *MALAT1*-inhibition on *MAPK*-pathway genes demonstrated the important regulatory process in melanoma. The results of Feichtenschlager et al. showed that *MALAT1*-ASO treatment decreased *BRAF* RNA expression and protein levels. Moreover, *MALAT1* had an increased correlation with *MAPK*-pathway-associated genes in melanoma samples compared to healthy skin (14). *MALAT1* regulates miRNA-34a, targeting *c-Myc* and *Met* transcripts. Specifically, *MALAT1* regulates miRNA-34a stability in melanoma cells. Importantly, *MALAT1* was significantly enriched in the Ago2 complex, but not when the *MALAT1*-binding site of miR-34a was mutated. *MALAT1* levels were markedly higher in melanomas than in paired adjacent normal tissues. *MALAT1* was also identified as a competing endogenous RNA (i.e., miRNA-183 sponge). Furthermore, *MALAT1* promotes melanoma cell growth and invasion by silencing miRNA-140 or miRNA-22 (96).

Within the *MAPK* axis, there are additional regulators in melanoma. Knowing that *RAF*-dependent *MAPK1* mutations appear alongside *MAPK* pathway mutations, contributing to aggressive melanoma behavior (97), another association was identified. *LINC01296* regulates the miRNA-324-3p/*MAPK1* axis in CMs. The rescue experiments reported by Wang suggested a positive regulatory association of *LINC01296* with *MAPK1* and that *LINC01296* regulates miRNA-324-3p in melanoma (24). However, the role of *MALAT1* in the regulation of gene and protein expression of the *MAPK*-pathway kinases RAS, RAF, MEK, and ERK in melanoma is still largely unknown.

The sex-determining region Y (SRY)-related high-mobility group (HMG) box (SOX) family consists of 20 transcription factors. The dysregulation of *SOX* family members is associated with tumorigenesis, metastasis, enhanced cell proliferation, EMT, stemness, and drug resistance in almost all cancers, including melanoma (98). Despite considerable research to investigate the mechanisms and functions of the *SOX* family, melanoma remains a tumor in which *SOX*-family involvement has many more details to uncover. Therefore, the gene that encodes for lncRNA, *LINC01063*, was found highly expressed in advanced melanoma stages, functioning as a sponge for miRNA-5194 to upregulate *SOX12* expression (19). *SOX12* expression can be used as a diagnostic and prognostic biomarker in CM (99).

Earlier studies have shown *SOX4* to be involved in melanoma cell migration and invasion (100). More recently, lncRNA *MEG3* was shown to sponge miRNA-208, further silencing *SOX4*. Therefore, downregulation of lncRNA *MEG3* is associated with poor clinical outcomes in CM patients. Overexpression of lncRNA *MEG3* inhibited cancer stem cell differentiation and suppressed melanoma progression and metastasis (32). These are examples of axes comprising miRNAs and the phenomenon of sponging by lncRNA that affects a specific target, in our cases, members of the *SOX* transcription family. These axes can constitute melanoma biomarkers for prognosis or even future therapy targets.

Another example in the search for new therapeutic approaches based on lncRNA and their targets involves signal transducer and activator of transcription (STAT) proteins. The STAT family is a group of transcription factors critical for both tumorigenesis and immune responses. STAT3 and STAT5 cascades are commonly activated during melanoma progression and mediate metastatic processes. Deactivation of these cascades enhances antitumor immune responses, is efficient against metastatic melanoma in the preclinical setting, and emerges as a promising targeting strategy, especially for patients resistant to immunotherapies (101). KCNQ1 overlapping transcript 1 (*KCNQ1OT1*) sponges and hinders the effect of miRNA-34a, which has *STAT3* as a downstream target. This phenomenon induces transcriptional regulation of programmed cell death 1 ligand 1 (*PD-L1*). Knocking down *KCNQ1OT1* reduced *PD-L1* levels and enhanced CD8+ T-cell cytotoxicity against melanoma cells. The miRNA-34a/*STAT3/PD-L1* axis promotes immune evasion of melanoma cells, pointing to a potential therapeutic target in CM (27).

Cyclin-dependent kinase 16 (CDK16) is involved in the cell cycle, vesicle trafficking, spindle orientation, and many other physiological cellular processes. In pathology, it is involved in tumor cell growth, proliferation, and metastasis (102). In melanoma, *CDK16* is overexpressed and regulated by miRNA-324-5p, which is sponged by HCG18 (28). Therefore, the HCG18-miRNA-324-5p couple could be a potential treatment target and possibly constitute diagnostic markers in CM. miRNA-324-5p is not the only miRNA regulating *CDK16*; miRNA-33a is another regulator of this cyclin kinase (103). miR-33a is sponged by *CCAT1*. The expression of *CCAT1* was found to be significantly upregulated in melanoma tissue and cell lines, and *CCAT1* knockdown suppressed proliferation, migration, and invasion (104). The presented examples underline the fact that specific molecules and processes within melanoma tumorigenesis are regulated by several miRNAs, which are also regulated by different lncRNAs. Most likely, these cross-functions were developed as part of a tumor evasion strategy to hinder and divert the host’s antitumor actions.

Emerging evidence validates the critical role of lncRNAs in melanoma development through mechanisms that rely on the sponging phenomenon. LncRNAs and their interaction with miRNAs have a dual function. LncRNAs can be both tumor suppressors and oncogenes, competing with miRNAs and influencing the translational process.

#### 2.3.2 circRNAs

Circular RNAs (circRNAs) are other members of non-coding RNAs that may act as sponging molecular regulators of miRNAs, playing crucial roles in the biology of various cancers. CircRNAs are abundantly expressed endogenous molecules. Apart from functioning as miRNA sponges and regulating the proliferation, differentiation, and invasion of cancer cells, circRNAs can sequester RNA binding proteins (RBPs) and even translate into proteins (105).

In melanoma, circRNAs are still to be explored as they are documented to be linked mainly to melanoma progression, and some examples will follow. *CDR1* is one of the best-studied circRNAs in melanoma; it harbors over 70 conserved binding sites for miRNA-7 (106). miRNA-7 is a tumor suppressor in melanoma, repressing proto-oncogenes such as *EGFR*, *IGF1R*, and *PIK3CD* (107).

Like lncRNAs, circRNAs also regulate important signaling pathways in melanoma. For example, Yang et al. have shown that *circFCHO2*(*hsa_circ_0002490*) is consistently overexpressed in melanoma tissues, correlated with a poor prognosis for patients. This circRNA has fewer miRNA binding sites and more RBP sites. The study shows that *circFCHO2* determines melanoma progression by regulating the *PI3K/AKT* signaling pathway through binding to dead end protein homolog 1 (*DND1*) (33), and thus, it can be a potential diagnostic biomarker in CMs.

In melanoma progression, *circRTTN*, miRNA-890, and EPH receptor A2 (*EPHA2*) were examined in various models. *CircRTTN* and *EPHA2* were found to be upregulated, while miRNA-890 was downregulated. *CircRTTN* was identified as an effective molecular sponge for miRNA-890, negatively regulating its expression. Thus, *circRTTN* mediates melanoma progression by regulating the miRNA-890/*EPHA2* axis (34). *CircFAT1* and miRNA-375 have another functional relationship in melanomas. *CircFAT1* is significantly overexpressed, while miRNA-375 expression is underexpressed. Mechanistically, *circFAT1* positively regulates *SLC7A11*, an important transporter of cystine into cells, maintaining cellular redox balance and glutathione synthesis. This effect is achieved by sponging miRNA-375, which allows tumor cells to actively proliferate and invade (35). *Hsa_circ_0103232* was reported as a functional sponge for miRNA-661, increasing the expression of *RAB3D*, a gene encoding a GTPase that regulates intracellular membrane trafficking. In melanoma tissues, *hsa_circ_0103232* expression was negatively correlated with miRNA-661 and positively correlated with *RAB3D*. Therefore, *hsa_circ_0103232* is a tumor promoter via sponging miRNA-661/*RAB3D* (36). The expression of *circRPS5*, miRNA-151a, and *NPTX1* was studied in melanomas, and it was reported that *circRPS5* is significantly downregulated, acting as an miRNA sponge and providing a promising therapeutic strategy for melanoma (37).

A different effect was reported in melanoma with *circITCH* expression, which was found to be repressed in melanoma *versus* normal tissues. This molecule, *circITCH*, downregulates melanoma proliferation and metastasis, a mechanism sustained by the sponging of miRNA-660 to upregulate *TFCP2*, a known suppressor of melanoma progression (38). **Table 1** presents a list of the major sponging molecules for mRNAs specific to melanoma tissue and their clinical relevance.

## 3 miRNAs in liquid biopsies

The gold standard in CM diagnosis is excisional biopsy with complete resection or incisional tissue biopsy. However, being invasive, these biopsy techniques are not suitable for long-term disease monitoring (108). Liquid biopsy (LB) represents a non-invasive, substantial, and modern tactic in melanoma early detection, as tumor-specific components (e.g., ctDNA, miRNAs, and CTC) released into biological fluids provide additional information useful for diagnosis, treatment decisions, and risk assessment. Significantly less invasive than tissue biopsy, LB represents a new approach to monitor treatment response and disease recurrence, predicting metastasis. LB is a rich source of tumor components and represents a refined tool for providing information by either monitoring a single marker or a combination of markers to enable advanced disease detection. Epigenetic modifications, chromosomal aberrations, along with the failure of transcriptional control mechanisms, cause dysregulation of miRNA expression in CM. miRNAs occur as free molecules, packed in exosomes, or in complexes with proteins and lipids. Due to packaging *into* extracellular vesicles (EVs) when secreted by melanoma cells, EV-derived miRNAs are protected from RNase action and are thus extremely suitable for intense research. The presence of specific miRNAs and their levels are dependent on cancer type or other parameters like treatment response (109).

### 3.1 Biological sources for miRNAs as circulating biomarkers

miRNAs can be detected in a variety of body fluids such as serum, plasma, urine, CSF, saliva, but also in cell-conditioned media. Based on the release mechanism, circulatory miRNAs can be subdivided into two major categories: non-vesicular (cell-free) and EV-derived miRNAs, with certain representatives selectively exported in exosomes (110).

The properties of different circulating miRNAs have been studied in close relation to their biogenesis. miRNA biogenesis is an ordered two-phase process, initiated in the nucleus and completed in the cytoplasm. Mature miRNAs are yielded from irregular hairpin structures that are specifically processed by the RNAase III enzymes Drosha and Dicer. These ordered cleavages give rise to single-stranded molecules that are exported from the nucleus into the cytoplasm and loaded into the RNA-induced silencing complex (RISC) to fine-tune gene expression by binding to the 3’ untranslated regions (3’UTRs) of target mRNAs (miRISC). This defines the miRISC-related canonical miRNA biogenesis pathway (111). Hence, upon their release into circulation, non-vesicular miRNAs are protected from degradation through their association with the argonaute proteins (AGO) of the RISC complex or with other RNA-binding proteins. Regarding EV-associated miRNAs, at least three EV types (exosomes, microvesicles, and apoptotic bodies) have been described based on their genesis, size, and function, which will be presented in detail in Section 3.1.2 (112, 113). A rough distinction between cell-free miRNAs and vesicular miRNAs can be made by RNase treatment, which facilitates the removal of unprotected miRNAs; however, it should be noted that this treatment is not effective in discriminating between AGO-complexed cell-free miRNA and exosomal miRNA (114).

#### 3.1.1 Circulating free miRNAs

Although it was long believed that EV-associated miRNAs were the most abundant molecules in biological fluids, studies have dispelled this myth, demonstrating that 90%–99% of circulating miRNAs exist in a vesicle-free state. These cell-free miRNA molecules are stabilized, both in plasma and in cell culture-conditioned media, solely through complexing with different RNA-binding proteins such as the AGO family proteins or high-density lipoprotein (HDL), which are highly effective in protecting them from environmental nucleases (115). Furthermore, the presence of circulating miRNAs free from RISC raises some questions regarding the potential miRNA exchange between individual RISCs and the additional factors that may recognize and stabilize miRNA molecules in body fluids (116).

#### 3.1.2 EV-associated miRNAs

Interestingly, the miRNAs enclosed in EVs represent just a small fraction (1–10%) of the total pool of circulating miRNAs. EVs are lipid bilayer-enclosed particles encapsulating a plethora of molecular constituents, such as proteins, lipids, DNA, and heterogeneous species of RNA derived from the parent cell, including miRNAs. In recent years, various EV biogenesis mechanisms have been described, involving, among others, members of the ESCRT system or the Ras family of small GTPases, tetraspanins (CD9, CD63, and CD81), or the depolymerization of the actin cytoskeleton (117). Thus, based on their biogenesis route and size, EVs have been subdivided into three main classes: microvesicles (0.1–1 µm; derived from the plasma membrane), exosomes (50–200 nm; originating from the endosomal pathway), and apoptotic bodies (1–5 µm; generated by apoptosis) (118, 119). Specific surface markers have been described for all EV classes, including CD9, CD63, Alix, and TSG101 for exosomes; integrin, selectin, and Annexin V for microvesicles; and DNA, histones, and Annexin V for apoptotic bodies.

The mechanisms by which miRNAs are incorporated into EVs and exosomes, respectively, have not been fully elucidated; however, four possible pathways have been described (1): the miRISC-dependent canonical pathway (2); the endosomal sorting complexes required for transport (ESCRT)-independent, ceramide-mediated pathway (3); the sumoylated heterogeneous nuclear ribonucleoproteins (hnRNPs)-related pathway; and (4) the 3’end uridylation signal pathway (113).

miRNAs are stable in LBs, especially when loaded in EVs, thus being promising candidates for melanoma biomarker panels. Moreover, EV-derived miRNAs play key roles in intracellular communication, as they can be readily internalized by neighboring cells through endocytosis, where they can modulate a wide range of biological processes with great impact on the TME (113). This property of EVs is currently being exploited for their use as drug delivery vehicles within the TME.

### 3.2 Circulating miRNAs as diagnostic/prognostic tools in skin melanoma

#### 3.2.1 Cell-free miRNA signatures

The miRNA stability is a crucial advantage for their use as biomarkers in LB. Various expression levels of selected miRNAs in melanoma patients could represent superior biomarkers. Moreover, a combination of miRNA, ctDNA, and CTCs would allow for better melanoma clinical management. Technically, an adequate miRNA biomarker armamentarium should emerge from a proper amplification method. However, variations in the sensitivity and specificity of qRT-PCR assays associated with differences in miRNA extraction kits could generate contradictory results. For example, an increased serum miRNA-16 level has been reported in melanoma patients compared to healthy controls (120). Meanwhile, an interesting finding is that a decrease in serum miRNA-16 levels occurs as melanoma progresses to advanced stages, being strongly correlated with tissue expression of Ki-67 (120, 121).

By analyzing the plasma expressions of hsa-miR-200c-3p, hsa-miR-144-3p, and hsa-miR-221-3p using qPCR, a differential expression was revealed in melanoma patients *versus* controls. Hsa-miR-221-3p acts as an oncogene, while hsa-miR-200c-3p and hsa-miR-144-3p are onco-suppressors. In addition, hsa-miR-144-3p and hsa-miR-221-3p have proven relevant for the early diagnosis of melanoma, while hsa-miR-200c-3p may be useful for monitoring high-risk stages. The co-expression of these three miRNAs has been shown to be a promising biomarker for differentiating benign nevi from melanoma. In patients with *BRAF* mutations, serum levels of the tumor-suppressive miRNAs miRNA-579-3p and miRNA-4488 could predict progression-free survival of metastatic melanoma patients, independently of serum LDH levels, indicating these miRNAs as independent biomarkers (122, 123).

Despite their increased accessibility and stability in LBs, the clinical value of cell-free miRNAs across various biological fluids and challenging settings (such as brain metastasis) has not been well examined. For this reason, a recent study investigated the diagnostic relevance of miRNA profiles in histopathologically confirmed BM melanoma metastases, corresponding plasma samples, and CSF samples in patients with different types of cancer, including CM. Significantly differentially expressed miRNAs were observed in BM tissues compared to primary tumors. While tissue-specific microRNA signatures showed the highest accuracy, these findings also highlight the low diagnostic utility of circulating miRNAs in patients with BM. Plasma samples revealed a greater number of differentially expressed miRNAs that overlapped with tissue signatures; however, the identified signatures showed limited prognostic value due to contamination with non-specific miRNAs derived from other cell types, which can significantly impact the detection of low-abundance miRNAs (58).

#### 3.2.2 EV-derived miRNA signatures

In several solid malignancies, such as breast, lung, liver, and prostate cancers, the diagnostic and prognostic value of EV-derived DNA fragments, proteins, and miRNAs has been established (117). However, in CM, therapeutic efforts with EV-miRNA profiling are currently the subject of intense investigation, and research advances are being made more toward evaluating the proteome of EVs. Thus, evaluating the proteomic profile of melanoma cell-derived small EVs in plasma revealed a distinctive eight-protein signature able to differentiate patients with progressive disease from those with non-evident or stable disease (124), suggesting prognostic relevance for melanoma-derived EV profiling (125, 126).

Some recent reports also support EV-derived miRNAs as very promising biomarkers for melanoma progression. Thus, by analyzing plasma EVs from patients with metastatic melanoma, a signature of miRNAs with differentiating power between melanoma patients and controls was identified. Hence, four miRNAs (miRNA-412-3p, miRNA-507, miRNA-1203, and miRNA-362-3p) were proposed as a non-invasive tool for the diagnosis and prognosis of CM (104). Another recent report confirmed that increased levels of miRNA-92b-3p, miRNA-182-5p, and miRNA-183-5p endorse these miRNA-EVs as potential biomarkers for melanoma diagnosis (127).

#### 3.2.3 Exosomal miRNA signatures

Our recent understanding of the role of exosomal miRNA (Exo-miRNA) in modulating the tumor environment emerges as a prospective option for cancer therapy (114). Exosomes protect miRNAs from degradation and originate from both tumor cells and immune cells, thereby reflecting the interaction between the tumor and the host. Analysis of plasma-derived exosomes can reveal accurate miRNA signatures when comparing patients with healthy subjects or when comparing different stages of melanoma (128). Moreover, exo-miRNAs can serve as biomarkers to differentiate early-stage melanoma from advanced-stage patients. Thus, an analysis of an adequate number of samples revealed that a panel consisting of Exo-miRNA-532-5p and Exo-miRNA-106b identified melanoma patients with a sensitivity of 92.0% and 88.0% for healthy subjects, indicating Exo-miRNAs as a helpful tool in the detection, prognosis, and therapy of melanoma (129).

In another study, exosomal miRNA profiling of CM patients’ plasma revealed a decreased miR-1180-3p expression in comparison to healthy subjects, emphasizing a potential melanoma diagnostic biomarker. The diagnostic efficiency of Exo-miR-1180-3p in melanoma was assessed in the validation groups of patients and controls. Moreover, the level of Exo-miR-1180-3p was negatively associated with the proliferation, migration, and invasion of melanoma cells, underlying Exo-miR-1180-3p as a promising diagnostic marker in melanoma (130).

Finally, by exploring a panel of miRNAs composed of hsa-miRNA-149-3p, hsa-miRNA-150-5p, hsa-miRNA-21-5p, hsa-miRNA-200c-3p, hsa-miRNA-134-5p, hsa-miRNA-144-3p, and hsa-miRNA-221-3p in plasma-derived exosomes, it was found that three miRNAs of the panel—hsa-miRNA-200c-3p, hsa-miRNA-144-3p, and hsa-miRNA-221-3p—were differentially expressed in melanoma patients compared to the control group. This differential triad miRNA expression may complement routine histopathological practices in CM, providing a non-invasive approach for discriminating between nevi and melanoma in clinical cohorts (131).

### 3.3 Circulating miRNAs as biomarkers and key determinants of therapeutic responses in CM

#### 3.3.1 EV-derived miRNAs may unravel novel therapeutic targets in melanoma

CM is one of the most immunogenic human tumors, with a high mutational burden that results in a diverse collection of neoantigens. These observations have been exploited for the development of revolutionary immune checkpoint inhibitors (ICIs), such as those targeting the PD-1/PD-L1 axis, which have significantly improved patient survival rates. At the same time, melanoma remains a highly immunosuppressive and heterogeneous tumor, in which various components of the TME interact closely to facilitate tumor progression, immune evasion, and therapy resistance. Therefore, a better understanding of miRNA-modulated mechanisms associated with immunosuppression in CM may lead to innovative targeted interventions that may synergize with ICIs, aiming at superior prognosis and clinical outcomes in CM patients (132).

The role of EVs in the anti-tumor immune response against melanoma has been extensively studied since the implementation of ICI therapy (133). In a study similar to Andrade et al. (134), Huber et al. showed that melanoma EVs can mediate the transition of monocytes to immature myeloid-derived suppressor cells (MDSCs) (135). These cells had all the characteristics to hinder the immune response, namely, downregulating the expression of *HLA-DRA* (Major Histocompatibility Complex, Class II, DR Alpha), enhancing the transcription of *IL-6* and *CCL2*, and inhibiting the activity of T cells. When cells were incubated with plasma harvested from melanoma patients containing EVs, similar results were obtained (135). Further investigating the miRNA content of these EVs, Xiao et al. showed that EVs released from melanoma cells contain miRNA-191 and Let-7a. These miRNAs are involved in several pro-tumorigenic pathways, such as EMT switching of melanoma cells and loss of adhesion factors (136). Conversely, Ataollahi et al. have shown that miRNA-211 introduced into tumor-derived exosomes induced an anti-tumor immune response in experimental animal models. An increased survival rate, lower suppressive T lymphocytes in animal tumors, and higher effector T cells were registered (137).

Macrophages play essential roles in establishing an immunosuppressive tumor setting, and the interaction between tumor cells and tumor-associated macrophages (TAMs) is critical for tumor growth. By miRNA sequencing analysis, miRNA-708-5p was found to be abundant in melanoma exosomes, facilitating M2 polarization of macrophages, which in turn promoted melanoma progression and metastasis. At the macrophage level, miRNA-708-5p directly targets the transcription factor *FOXN3* and subsequently activates the *PI3K/AKT/mTOR* pathway (**Figure 4**). Downregulation of *FOXN3* by miRNA-708-5p impedes the phagocytic capacity of macrophages and augments the secretion of IL-10 and TGF-β. These data provide new insights regarding the possibility that miRNA-708-5p and *FOXN3* could be explored as therapeutic targets in melanoma (138).

**Figure 4:**
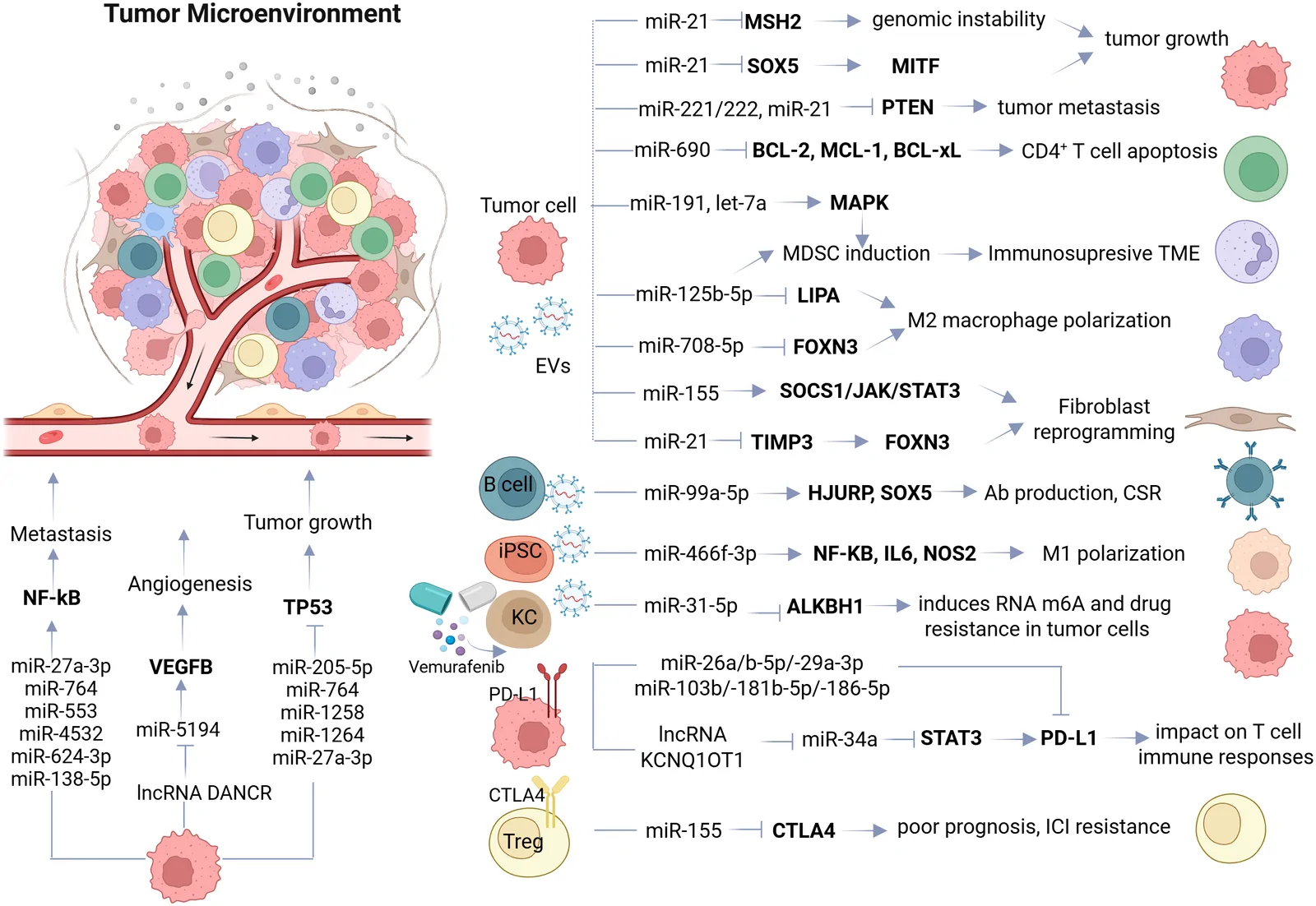
The miRNA-mediated crosstalk between tumor cells and other cellular populations within cutaneous melanoma (CM) microenvironment. The figure illustrates the most important miRNAs within the tumor microenvironment (TME) that have been associated with metastasis, immunosuppression, and response to immunotherapy and targeted therapies. Arrows indicate target activation, whereas the T-bars indicate downregulation. The presence of a vesicle next to a specific cell type indicates the vesicular origin of the indicated miRNAs, whereas the absence of the symbol indicates the tissue-related roles of miRNAs. MSH2, MutS Homolog 2; SOX5, -SRY-Box Transcription Factor 5; MITF, melanocyte inducing transcription factor; BCL2, BCL2 apoptosis regulator; MCL-1, MCL1 apoptosis regulator; BCL-xL, B-cell lymphoma-extra large; MAPK, mitogen-activated protein kinase; LIPA, lipase A, lysosomal acid type; FOXN3, forkhead box N3; SOCS1, suppressor of cytokine signaling 1; JAK, janus kinase; STAT3, signal transducer and activator of transcription 3; TIMP3, tissue inhibitor of metalloproteinase 3; HJURP, Holliday junction recognition protein; IL-6, interleukin 6; NOS2, nitric oxide synthase 2; ALKBH1, AlkB homolog 1, histone H2A dioxygenase; CTLA4, cytotoxic T-lymphocyte associated protein 4; CAF, cancer-associated fibroblast; MDSC, myeloid derived suppressor cells; KC, keratinocyte; iPSC, induced pluripotent stem cells; EVs, extracellular vesicles; PD-L1, programmed cell death ligand 1; Ab, antibody; CSR, class switch recombination; PTEN, phosphatase and tensin homolog; NF-kB, nuclear factor kappa-light chain enhancer of activated B cells; and VEGF B, vascular endothelial growth factor B. The figure was created in https://www.Biorender.com.

In the search for innovative immunoregulatory strategies for the eradication of metastases, the molecular mechanisms by which EVs could regulate macrophage functionality in the control of metastases were analyzed. Using a Rag2-deficient mouse melanoma liver metastasis model, it was observed that pre-treatment with induced pluripotent stem cell-derived EVs (iPS-EVs) significantly repressed liver metastasis, and the effect was prolonged. Furthermore, it was proven that macrophage depletion entirely abolishes the anti-metastatic effect, indicating macrophages as essential intermediates of the process. Moreover, miRNA profiling in iPS-EVs revealed a high abundance of miRNA-466f-3p, responsible for *NF-κB* activation and increased expression of *IL-6* and *Nos2* in macrophages. These discoveries suggest that miRNA-466f-3p is a pro-inflammatory activator of macrophages, making iPS-EVs a promising platform for EV-based immunotherapy aimed at modulating innate immunity to suppress tumor metastasis (139).

#### 3.3.2 Circulating miRNAs are linked with melanoma immunotherapy responses

##### 3.3.2.1 EV-derived miRNAs and melanoma ICI responses

Resistance to ICIs is one of the most complex and intensively investigated topics in melanoma. EVs can mediate drug resistance through direct drug sequestration, transfer of pro-survival proteins and RNA, induction of cancer stem cells, and interaction with cells of the TME and the immune system (140). Interestingly, under the effect of antitumor therapy, both sensitive (dying cells, senescent cells) and resistant tumor cells (e.g., cancer stem cells), as well as non-tumor cells of the TME, begin to produce different EVs in large quantities. A complex panel of EV-derived molecules is starting to be discovered (141), along with their involvement in drug resistance mechanisms. Taking into account the key role of EVs in melanoma pathogenesis and progression, as detailed in the preceding sections, and in light of their crucial involvement in several mechanisms of cancer drug resistance, EVs released by melanoma cells might play a role in the development of resistance, including against immunotherapeutics.

Briefly, miRNAs detected in EVs derived from melanoma cells appear to regulate immunotherapy responses through various pathways. For example, tumor-derived miR-125b-5p is considered to hinder the response of CM to ICIs by promoting the induction of MDSCs (135) and M2-TAM phenotypes (142). Furthermore, miR-690-enriched EVs released by melanoma cells have been shown to trigger mitochondrial apoptosis in CD4+ T cells, further promoting tumor progression (143). These observations are very important, as CD4+ T cells act synergistically with CD8+ T cells in antitumor immunity, and maintaining CD4+ T cell cytotoxic functions is essential for effective immunotherapy responses (**Figure 4**) (144). Conversely, B-cell-derived EVs have also been reported to provide non-invasive biomarkers for immunotherapy responses in CM. Specifically, the tumor suppressor miR-99a-5p was found to be enriched in CD19+ EVs of responders versus non-responders. It was further shown that miR-99a-5p is selectively encapsulated in CD19+ EVs in responding patients and plays a key role in B-cell-mediated antitumor activity. That is, functional analysis revealed that the downregulation of miR-99a-5p in B cells diminishes the antitumor effects of T cells, as this miRNA is essential for B-cell maturation via class-switch recombination (**Figure 4**) (145).

The role of some miRNAs in CM is still controversial, being both pro-oncogenic and tumor suppressors. For instance, exosomal miRNA-155 has been linked to ICI therapy resistance in melanoma, although a phase II study involving a miRNA-155 inhibitor (Cobomarsen) in melanoma was discontinued at the pharmaceutical company’s decision (146, 147).

In a 2026 study analyzing EV-associated miRNAs in the plasma of stage III-IV CM patients undergoing anti-CTLA-4 immunotherapy and guadecitabine (a DNA hypomethylating agent), a dynamic profile of various EV-miRNA molecules was identified. Before therapy, a complex EV-miRNA signature specific to non-responder (NR) patients was found (seven differentially expressed, three up-regulated, and four down-regulated miRNAs). Additionally, an EV-miRNA panel related to primary resistance to therapy was identified (miRNA-497-5p, miRNA-99a-3p, miRNA-592, and miRNA-548b-3p). Moreover, the authors assessed a therapy-modulated EV-miRNA signature, noting nine molecules (miRNA-150-3p, miRNA-576-5p, miRNA-26b-3p, miRNA-34a-5p, miRNA-556-3p, miRNA-1183, miRNA-1203, miRNA-604, and miRNA-892a) consistently deregulated at weeks 4 and 12 post-treatment. A signature of EV-miRNAs linked to resistance and poor survival was also identified. At week 12, 27 EV-miRNAs varied between NR and responders; specifically, miR-1203 and miR-556-3p were up-regulated in NR, with miRNA-1203 recognized as a diagnostic biomarker of CM (148). These findings highlight that an EV-miRNA signature can qualify as a non-invasive biomarker panel in predicting and following up on treatment (149).

##### 3.3.2.2 Cell-free miRNAs and melanoma ICI responses

Cell-free miRNAs have also proven helpful in predicting immunotherapy efficacy long before imaging confirmation in different cancer types (150). In particular, in CM, miRNA-21-5p and miRNA-146a-5p reveal specific dynamics during treatment, providing information on the emergence of response or the development of therapy resistance (151). Moreover, in patients with stage IV melanoma who progressed after receiving anti-CTLA-4 (ipilimumab), anti-PD-1 (vivolumab or pembrolizumab), or their combination, overexpression of miRNA-4649-3p and miRNA-615-3p has been observed (152).

#### 3.3.3 Circulating miRNAs regulate melanoma responses to targeted therapy

Circulating miRNAs have been recently investigated in relation to therapeutic regimens based on BRAF and MEK inhibitors, for which over 50% of melanoma patients are eligible. The goal is, of course, to identify novel molecules that can determine and predict disease progression in a timely manner, allowing for an effective change in therapy.

##### 3.3.3.1 EV-derived miRNAs and targeted therapy responses

It is known that keratinocytes influence the sensitivity of melanoma cells to vemurafenib, but a novel approach may be to dissect the role of keratinocyte-derived exosomes in melanoma cell sensitivity and how this might influence the therapy response. A recent study investigated the effects of keratinocyte-derived exosomes on melanoma cell growth and sensitivity to vemurafenib, corroborating the miRNA profiles of exosomes derived from the HaCaT keratinocyte cell line with the levels of miRNA-31-5p, AlkB homolog 1 (*ALKBH1*), and m6A RNA in A375 and UACC62 melanoma cell lines. The study data showed that vemurafenib treatment elevates miRNA-31-5p in keratinocyte-derived exosomes, inducing m6A RNA increment in melanoma cells, promoting melanoma proliferation, and reducing vemurafenib sensitivity. Therefore, a potential therapeutic strategy to increase vemurafenib sensitivity in *BRAFV600E* melanoma cells is to target the miRNA-31-5p/*ALKBH1* axis and modulate RNA m6A levels (153). Among the mechanisms underlying melanoma progression, tumor microenvironment remodeling plays an essential role in tumorigenesis. A key frontier in tumor mechanism research is identified with EVs that influence the microenvironment and modulate a variety of cellular mechanisms. In a study using two types of melanoma-derived cell lines—highly and poorly metastatic—it was demonstrated that the highly metastatic type can enhance the metastatic capacity of poorly metastatic cells by transferring EVs; these encapsulate functional miRNAs, including miRNA-23a-5p. Once entered into poorly metastatic cells, EV-miRNA-23a-5p induces changes in m6A methylation levels in the mRNA corresponding to the tumor suppressor genes *Mtus1* and *Prrg4*. This modulation subsequently influences the expression of these genes and, consequently, the invasive behavior of poorly metastatic cells (154).

##### 3.3.3.2 Cell-free miRNAs and targeted therapy responses

To establish if circulating miRNAs could be predictive biomarkers in CM, plasma miRNAs were profiled using next-generation sequencing at baseline and during the clinical evolution of patients receiving targeted therapy, BRAF inhibitors (BRAFi), or BRAFi + MEKi. Initial median values of miRNA-1246 and miRNA-485-3p were significantly lower in responder patients, with a tendency for miRNA-1246 levels to increase and miRNA-485-3p levels to decline at the time of disease progression. The study revealed that low miRNA-485-3p levels and a high miRNA-1246/miR-485-3p ratio were independent negative prognostic factors for PFS in multivariate analysis. Thus, profiling plasma levels of miRNA-1246 and miRNA-485-3p at baseline may identify CM patients who are likely to fail to respond to targeted therapy or who are at higher risk of short-term PFS and mortality, thereby improving their management (155).

However, other research groups have turned to testing these biomarkers on more sensitive platforms, such as digital PCR. In their previous reports on BRAF/MEK-targeted therapy responses in clinical cohorts, De Gregorio et al. identified miR-4488 as associated with tumor aggressiveness and poor prognosis and miR-579-3p as exhibiting tumor-suppressive activity in CM. Based on these findings, a novel duplex digital PCR approach was developed and validated for the simultaneous detection of miR-4488 and miR-579-3p in the serum of patients with *BRAF*-positive metastatic melanoma receiving MAPKi. The study designated the expression ratio between miR-4488 and miR-579-3p as “miRatio.” Researchers converted it into a biomarker that outperformed individual miRNAs and even LDH in predicting PFS in melanoma patients. Thus, this approach, based on the biological complementarity of two key miRNAs, demonstrates that miRatio is a dynamic biomarker that better captures the relationship between tumor progression and therapeutic response than individual miRNA measurements (156).

## 4 Concordance/discordance between tissue and circulating miRNAs

### 4.1 Concordance of miRNAs in tissues and liquid biopsies

In CM, concordance between tissue and LB investigation is needed for precise diagnostic and prognostic evaluation of clinical outcomes. According to recent studies, concordance reflects the detection of the same druggable alteration in both biopsy types (tissue and liquid/plasma), while discordance refers to clinically relevant markers detected by only one approach (157). Genomic discordance between tissue biopsy and LB can reach up to 50% (158) due to tumor heterogeneity, low ctDNA shedding (such as in brain metastases), and assay differences (NGS versus ddPCR), despite a 70–80% concordance for common driver mutations like *KRAS* or *BRAF* V600 (159). In addition, up to 50% of genetic alterations discovered in one biopsy type may be undetected by the other (160), and LB often detects emerging resistance mutations in multiple metastatic sites where a localized tissue biopsy fails (121).

In CM, there are several miRNAs whose expression remains concordant between tissue and plasma and which have demonstrated diagnostic and prognostic robustness in clinical settings. These include miR-21, miR-221, miR-222, miR-489, and miR-205, among others.

The oncogenic miR-21 is overexpressed in the tissues and sera of CM patients (109, 161, 162). It supports tumor invasiveness by targeting tumor suppressors such as *PTEN*. Serum miR-21 levels were effective in discriminating between melanoma patients and healthy subjects with 77% sensitivity and 82% specificity, and they correlated positively with the TNM stage (163). Tissue miR-21-5p levels were strongly associated with the American Joint Committee on Cancer (AJCC) stage, invasive depth, mitotic index, and lymphovascular invasion in clinical cohorts (164).

UV radiation exposure primarily induces the abundant production of circulating miR-21, including exosomal forms, across all major skin cell populations (melanocytes, keratinocytes, and fibroblasts). Various mechanisms by which exo-miR-21 supports melanomagenesis have been described (**Figure 4**). In addition to silencing *PTEN*, exo-miR-21 appears to drive this process by downregulating *SOX2*, an inhibitor of *MITF*. Furthermore, miR-21 targets *MSH2*, a key regulator of DNA mismatch repair, thereby increasing genomic instability in melanocytes, as well as *TIMP3* in fibroblasts, thus promoting an aggressive phenotype in the latter. Programmed cell death 4 (*PDCD4*), sprouty 1 (*SPRY1*), and cyclin-dependent kinase inhibitor 2C (*CDKN2C*) were also identified as additional miR-21 targets in melanoma cell lines. Conversely, miR-21 levels may be regulated by high-intensity focused ultrasound, subsequently reducing melanoma cell growth and invasiveness, thus pinpointing a potential strategy to mitigate the risk of secondary spread in CM (165).

Other oncomiRs that are abundant in CM tissues and circulation are miR-221 and miR-222 (166–168). They regulate *PTEN*, *p27*, and *c-KIT*, and converge toward the same signaling pathway (**Figure 4**) (169), being associated with aggressive CM features such as tumor thickness, poor differentiation, and reduced 5-year OS and disease-free survival rates (166, 170). Conversely, the tumor suppressor miR-489, which impacts melanoma glycolysis and proliferation, was downregulated in CM tissue and liquid biopsies, and it was associated with poor patient prognosis and advanced TNM stages (163, 171). miR-205 tissue and circulating levels were also reduced in CM patients, displaying a progressive decrease during the transition from benign lesions to metastatic melanomas (51, 172, 173).

miRNAs that show concordant expression in tissue and liquid biopsies are ideal for developing potential therapeutic strategies in dermato-oncology. For instance, lymphocyte-associated miR-150-5p, which was found to be downregulated in non-brisk tumor-infiltrating lymphocytes (TILs), *BRAF*-mutated (174), and brain-metastatic melanomas (175), may be restored to disrupt glycolytic processes and associated metastatic programs in tumor cells. This opens prospects for the adoption of miRNA mimics in CM treatment (176). This is consistent with findings that link elevated serum miR-150-5p levels to improved survival in patients with stage IV melanomas (174).

Concordant downregulation of tissue and circulating expressions (including exosomal) was also reported for the miR-193a arms. Induced expressions of miR-193a-3p and -5p species confirmed their tumor-suppressive roles, as a significant reduction in cell proliferation alongside reduced TROY, AKT/ERK, ERBB, and KRAS expressions was reported, emphasizing the therapeutic potential of these molecules in CM (177). Another relevant molecule is miR-204; its loss was reported in primary melanomas versus benign nevi, particularly in the *NRAS*-mutated subtypes. Its exogenous expression reduced the tumorigenic potential of melanoma cells, confirming previous observations from clinical cohorts regarding a potential correlation between elevated miR-204 levels and superior clinical outcomes (178). Additionally, targeted delivery of miR-204-5p and miR-199b-5p via lipid nanoparticles was able to abrogate the BRAFi-resistant phenotype by *VEGFA*, *TGFβ1*, *CCL5*, and *CXCL2* downregulation and impair tumor cell ability to recruit pro-tumoral M2 macrophages into the niche (12, 149, 179).

### 4.2 Discrepancies of miRNAs in tissues and liquid biopsies

A key discrepancy between tissue and circulating miRNA levels in melanoma stems primarily from biological keystones, in that both primary and metastatic tumors are distinct biological entities. Moreover, the activity of circulating biomarkers, including miRNA, strongly depends on specific environmental parameters, such as hypoxia, acidosis, and chronic inflammation (180). Additional factors such as host immune-inflammatory responses and co-existing pathologies may also significantly impact circulating miRNA levels in cancer patients (58, 181).

Selective packaging of certain miRNAs into EVs, RNase stability, and systemic clearance rates also account for the differential expressions of miRNAs across biological samples. Regarding the selective packaging of certain miRNAs into EVs, the precise reason for the preferential sorting of miRNA species into these vesicles remains unclear. RNA-binding proteins such as hnRNPA2B1 and Ago2 are the main players in this process, as they can specifically recognize the miRNA-binding motifs responsible for miRNA recruitment and sorting. However, in cancer, it is believed that the cargo transfer from parental cells to daughter cells is intended to drive disease progression and ensure the plasticity of the tumor niche. Conversely, other research groups suggest that this process represents merely a waste disposal mechanism, although it is highly plausible that cells within the TME may exploit this phenomenon for both waste elimination and signaling purposes (182).

Furthermore, an additional discordant element is the fact that tissue biopsies contain immune cells, fibroblasts, and normal keratinocytes that strongly distort the tissue miRNA profile, sometimes masking tumor-specific characteristics (168). A very modern approach regarding the spatial biology of tumor cells claims that heterogeneity within the melanoma tumor mass may explain why a small tissue biopsy may not correlate with systemic shedding patterns from various metastatic sites (161).

An additional data discordance may also arise from the multifaceted nature of miRNAs, which represent a complex class of biomarkers, with numerous families encompassing related species (e.g., hsa-miR-221-3p and 5p species), often with distinct targets and roles, unlike some established but “single” cancer biomarkers such as serum LDH (183). In this light, the evaluation of miRNAs as biomarkers in oncology starts from the premise of analyzing individual contributions within the TME, but their validation is often hampered by contradictory observations. A main cause of these inconsistencies is often attributed to the context-specific functions of microRNAs. Confusing nomenclature, involving the use of generic terms such as hsa-miR-211 rather than specifying the analyzed species, can also lead to profound discrepancies in miRNA level estimations across specimens. To allow a more harmonious interpretation of the apparently divergent data, both context-specific factors and fundamental concepts specific to microRNA biology must be analyzed. This perspective was exemplified by studying the role of miR-211-5p as a buffer against melanocyte state transitions, and it was revealed how the accurate estimation of domain-specific contexts is essential to advance toward a concordant understanding of miR-211-5p and other miRNAs in cancer biology. Therefore, it was underlined that miR-211-5p confers robustness to melanocytes, being a reliable nevus-related miRNA critical for target gene expression and phenotype switching in melanocytic cells. Yet, the genome, tumor microenvironment, and immune component of metastatic tumors are more heterogeneous compared to melanocytic nevi and primary cutaneous melanomas. Thus, although the phenotypic effects of miR-211-5p stated in these systems are variable, this may be due to a more difficult set of experimental conditions to outline (184).

## 5 Clinical translation, challenges, and future perspectives

### 5.1 Challenges and pitfalls in clinical translation

Despite the enthusiasm generated by recent advances in biomedical miRNA research, their clinical translation for diagnostic, prognostic, and predictive purposes has yet to be achieved for many pathologies. Circulating panels such as the miR Sentinel™ PCC4 Test and GASTROClear™ have received Food and Drug Administration (FDA) approval for clinical use in detecting prostate and gastric cancers. However, there are currently no clinically validated miRNA biomarkers for use in CM. Similarly, progress in evaluating miRNA-based therapies for cancer, including melanoma, has been modest, reaching only Phase I clinical trials (NCT01829971, NCT03884556). This contrasts with the field of siRNAs, where drugs such as STP707 have received FDA Investigational New Drug (IND) status for non-melanoma skin cancer, and STP705 has advanced to a Phase II clinical trial, demonstrating a 100% complete response (CR) rate in non-melanoma skin cancers (184, 185). Given that miRNAs and siRNAs share similar pharmacological properties, it appears that the major obstacle in translating miRNAs into clinical practice lies not necessarily in the sensitivity of these molecules, but rather in the inconsistency of their evaluation.

Beyond the aforementioned discrepancy in miRNA expression between liquid biopsies (LBs) and tissue biopsies, driven primarily by the preferential sorting of specific miRNAs into exosomes, the lack of reproducibility that has hindered circulating miRNA implementation in clinical settings is largely attributable to pre-analytical factors. Variables such as the sample type (fresh blood, serum, or plasma), collection tubes, the time elapsed between blood draw and processing, and the chosen analytical techniques (e.g., qRT-PCR, ddPCR, microarray, whole or targeted NGS), combined with cohort heterogeneity (differences in subtype, stage, and systemic history), significantly impact the accuracy of miRNA quantification in biological samples. Further complicating matters are issues regarding EV separation, RNA extraction techniques, the choice of endogenous controls for qRT-PCR data normalization, and the diversity of statistical methods employed (161). Collectively, these factors prompted the National Cancer Institute (NCI) to develop guidelines aimed at mitigating such biases and facilitating standardization in the field of circulating miRNA evaluation (186).

However, not all obstacles related to accurate profiling of circulating miRNAs have been overcome. Thus, despite the frequent use of endogenous controls such as U6, let-7a, or miR-1228, it must be noted that their expression varies dramatically across human tumors. Consequently, they may not be reliable for normalization in all pathologies. Therefore, it is recommended to include multiple reference genes and combine endogenous controls with exogenous ones (e.g., synthetic non-primate miRNAs like *cel-miR-39*) to control for technical bias during the analytical and post-analytical phases of testing (187).

Moreover, blood cells continuously release EVs and miRNAs into the circulation; thus, improper blood processing can lead to cellular contamination (e.g., with platelets) and hemolysis of the plasma or serum, thereby distorting the miRNA signatures detectable in these biological samples. Significant differences in circulating miRNA levels have also been reported between plasma and serum. For instance, the erythroid-specific miRNA (miR-451) was found to be more abundant in plasma than in serum compared to other miRNAs, proving that different plasma factors can significantly alter miRNA levels. In line with these observations, the use of membrane filtration combined with an RNase inhibitor is highly recommended for an accurate determination of miRNA signatures in patient plasma (188).

Confusing nomenclature, involving the use of generic terms such as hsa-miR-211 rather than specifying the analyzed species (-5p or -3p), which can have different biological roles, may also hamper the clinical translation of miRNA members in a clinical setting (131).

In cancer, miRNA-based therapeutic strategies aim to restore tumor-suppressive miRNA function (miRNA mimics) or inhibit oncogenic miRNAs (miRNA inhibitors such as anti-miR oligonucleotides, miRNA sponges, or decoys) to correct altered cellular processes. Despite promising results obtained in preclinical settings, miRNA-based drugs have not progressed beyond Phase II in clinical cohorts for various reasons related to efficiency, tolerability, delivery strategies, or related costs.

Silencing onco-miR-21 has proven highly effective in blocking tumor growth across multiple experimental models and in resensitizing breast cancer tumors to doxorubicin; however, anti-miR-21 strategies have not yet entered clinical trials for cancer, as a more detailed evaluation of their safety and tolerability in human subjects was required (NCT02855268) (189). A Phase 2 clinical trial evaluating Cobomarsen (SOLAR, NCT03713320), a synthetic locked oligonucleotide inhibitor of miR-155, was discontinued due to financial reasons despite very good tolerability profiles and a relative improvement in progression-free survival rates compared with vorinostat in patients with cutaneous T-cell lymphoma. In addition, a follow-up study (NCT03713320) was also terminated due to insufficient patient enrollment (190). Another promising candidate, MRX34, a liposomal miR-34a mimic, demonstrated good efficacy in limiting disease progression in several patients with primary liver cancer and other solid tumors in a Phase I clinical trial (NCT01829971); however, its evaluation was discontinued due to multiple immune-related adverse effects, such as hypoxia, hepatic failure, sepsis, and cytokine release syndrome (191). Therefore, issues involving immune responses, off-target effects, and production costs remain among the most significant barriers against synthetic miRNA drug translation into clinical practice. Compounding this is the fact that most studies evaluating miRNA-related mechanisms have been conducted *in vitro* or in different experimental models, including non-human primates that do not fully replicate the complexity of the human immune system (191).

Moreover, using a specific miRNA as a therapeutic target in CM requires great caution, as cellular miRNA levels are highly sensitive to environmental signals and can exert conflicting functions depending on the specific stressors and tissue or cellular compartments involved. For instance, elevated miR-146a-5p is a signature of melanoma brain metastases detectable simultaneously in cell lines, tissue biopsies, and exosomes. Inhibiting it impairs CM invasiveness via Notch dysregulation (192). Nevertheless, in the context of BRAF and MEK inhibitor exposure, reduced miR-146a-5p expression may be a hallmark of a drug-resistant phenotype due to its ability to regulate pro-survival *NF-kB* and alternative escape pathways (193). In the context of immunotherapy, miR-146a-5p disrupts the *STAT1/IFNγ* axis, leading to immunosuppression, whereas its inhibition synergizes with anti-PD-1 immune checkpoint blockade *in vivo*, resulting in superior outcomes compared with anti-PD-1 treatment alone (194). Another context-dependent miRNA is the immune-related, hypoxia-responsive miR-155 (195). The current literature indicates an onco-suppressive role for miR-155 in melanoma, as its downregulation was reported in CM patients versus normal controls. However, the deconvolution of immune populations within the TME revealed antigen dose and affinity as key determinants of miR-155 expression in CD8+ T cells. Anti-PD-1 therapy triggered an increase in miR-155 levels in tumor-infiltrating CD8+ T cells, which correlated with improved antitumor responses and enhanced immune infiltration in the lymph nodes of B16 melanoma mouse models. The same study reported elevated miR-155 in CM patients with superior outcomes (196). In contrast, other studies advocate for a pro-tumorigenic role in the CM microenvironment. Briefly, it was shown that melanoma cells may release miR-155-5p-enriched exosomes that can reprogram normal fibroblasts into pro-angiogenic cancer-associated fibroblasts (CAFs) via *SOCS1/JAK/STAT3* modulation (197). miR-155 was also found to be overexpressed in the Tregs of metastatic CM patients, where it downregulates *CTLA4*, leading to a poor prognosis (**Figure 4**). Taken together, all these mechanisms highlight the complex and multifaceted roles of miR-155 within the tumor microenvironment, underscoring the need to dissect the role of a specific miRNA across all major TME cell populations before targeting it or developing it into a biomarker (198).

### 5.2 Future perspectives in the field

The discontinuation of miRNA drugs in clinical trials has provided lessons that could open new perspectives for more effective pharmacological approaches in the field. Therefore, optimizing the miRNA delivery route is crucial for mitigating immunogenicity and the associated adverse effects of miRNA therapy. Direct injection of miRNA into the body causes single-stranded molecules to be perceived as pathogens, triggering a TLR-mediated innate immune response associated with the abundant secretion of inflammatory cytokines and type I interferons (199). Viral miRNA delivery systems, such as adenoviruses and lentiviruses, may also exhibit increased toxicity and immunogenicity, making non-viral delivery methods, such as liposomes and nanomaterials, preferred choices. Nanoparticles can also be recognized as foreign bodies and elicit immune responses; however, coating these particles with biocompatible materials, such as polymers, can reduce their immunogenicity (200). Additionally, natural EVs are emerging as promising drug delivery systems as they display good biocompatibility and increased potency to cross physiological barriers. Locked nucleic acid (LNA)-based synthetic miRNA therapies may also trigger an innate immune response when administered at high doses (201). Nonetheless, chemical modification of RNA molecules, leveraging nucleoside base modifications such as pseudouridine (Ψ) and N6-methyladenosine (m6A), has been shown to significantly reduce the immunogenicity of endogenous RNAs, thereby increasing the likelihood of translating miRNA drugs into the clinic. Although these modifications are particularly suitable for long linear RNA molecules, current efforts aim to apply them to molecular sponges as well. For instance, a recent report described how the immunogenicity of a circRNA containing two binding sites for miR-21, one for miR-221, and one for miR-195, was considerably reduced through 30% Ψ incorporation, without interfering with the inhibition of the target miRNAs (202).

In addition to low immunogenicity, controlled and targeted miRNA delivery is required to enhance clinical benefits and minimize side effects in cancer. For this reason, current efforts in the field are focused on designing systems capable of releasing miRNA drugs in response to variations in pH, temperature, enzyme levels, and redox potential within the target compartment (200).

Furthermore, the fact that a single miRNA has the capacity to fine-tune the expression of multiple genes (e.g., miR-28 regulates *BTLA*, *PD-1/PD-L1*, and *TIM-3* in melanoma) increases the likelihood of off-target effects that could disrupt unknown genes and networks, potentially leading to detrimental consequences. Therefore, a thorough functional characterization of each miRNA is essential to better understand its regulatory activities across multiple layers (200, 203). Recent progress in bioinformatics, which integrates network theory and artificial intelligence into prediction workflows, holds promise to mitigate off-target effects, leading to a more precise identification of miRNA candidates for targeted interventions in oncology (204, 205).

Despite the slow progress toward clinical applications, the benefit of miRNA assessment in tissue and LBs remains undeniable, as their dysregulation provides valuable insights into disease progression and therapeutic responses. Recognizing their significant clinical value, the scientific community has begun taking concrete steps to standardize biological sample collection and preservation methods, as well as protocols for EV isolation, miRNA extraction, and profiling. There is also a recognized need to validate the reproducibility of specific molecular signatures across large clinical cohorts. Furthermore, recent advances in single-cell and spatial sequencing techniques hold promise for a better understanding of the specific roles of miRNAs within different immune cell subsets (89). Thus, integrating new “omics” technologies and standardizing methodologies are expected to bring the clinical implementation of miRNAs for the diagnosis, prognosis, and monitoring of cancer patients, as well as for personalized therapy, one step closer to reality.

## 6 Conclusions

CM remains a leading cause of skin cancer-related mortality, with its incidence continuing to rise. Prognostic tools offer important clinical guidance but fail to capture the biological heterogeneity of CM or predict responses to therapies. Recent advances in prognostic and predictive molecular biomarkers have shown that tissue-based and circulatory molecular markers, such as miRNAs, can enhance risk stratification and orient therapy strategies. Well-known signaling pathways involved in the malignant transformation of normal melanocytes to malignant melanoma are regulated by a large panel of miRNAs that are themselves mainly regulated by lncRNAs and circRNAs. Current evidence supports miRNAs as promising diagnostic, prognostic, and predictive biomarkers in CM. The domain that remains to be pursued is the standardized methodologies and large-scale validation for integration into routine clinical practice.

Together, these advances reflect a shift toward precision oncology in CM, with the integration of biomarker-driven strategies that improve patient outcomes (206). The study of miRNAs and mRNAs as deep-regulators of transcription could open the clinical application of mRNA-based vaccination in melanoma (207), as the mRNA platform is already following the success of COVID-19 mRNA vaccines (208, 209).

There is a growing body of evidence, with additional data being gathered in real-time regarding miRNAs as emerging biomarkers in CM, highlighting their possible value in early detection, prognosis, and prediction of therapeutic response. Translational challenges and future directions for integrating ncRNA-based assays into clinical practice are demanding endeavors for transforming miRNAs into an accurate tool in precision medicine for melanoma management (210, 211).
